# Triage skill and associated factors among emergency nurses in Addis Ababa, Ethiopia 2017: a cross-sectional study

**DOI:** 10.1186/s13104-018-3769-8

**Published:** 2018-09-10

**Authors:** Sitotaw Kerie, Ayele Tilahun, Alemnesh Mandesh

**Affiliations:** 1 Department of Nursing, College of Medicine and Health Sciences, Bahir Dar University, Bahir Dar, Ethiopia; 2 Department of Nursing, College of Health Sciences, Mizan Tepi University, Mizan Teferi, Ethiopia; 3 Addis Ababa University College of Health Sciences, School of Allied Health Sciences, Addis Ababa, Ethiopia

**Keywords:** Triage, Skill, Associated factors, Nurse, Ethiopia

## Abstract

**Objective:**

The aim of this study was to assess levels of triage skill and associated factors among emergency nurses in Addis Ababa, Ethiopia, 2017.

**Results:**

Above half of the participants (52.9%) had a moderate level of triage skill. A strong positive relationship was found between nurses’ level of triage knowledge and skill (r = .68, p .01). Knowledge about triage, educational level and training experience had a significant relationship with triage skill with (B = 1.09, CI (1.41, 1.77), p = .002), (B = − 19.96, CI (− 30.208, − 9.715), p = .001), (B = .55, CI .16, .94), p = .006) respectively. This study revealed that most triage nurses had a moderate level of skills. Therefore, the ministry of health and hospitals should provide training and education to improve triage skill.

**Electronic supplementary material:**

The online version of this article (10.1186/s13104-018-3769-8) contains supplementary material, which is available to authorized users.

## Introduction

Triage is putting the patient in the right place at the right time to receive the right level of care, the allocation of appropriate resources to meet the patient’s medical needs. It also allows for the allocation of the patient to the most appropriate assessment and treatment area [1].

The triage system varies from one health institution to the other based on available medical services, community need and load of emergency departments [2].

Making nurse triage is not as such simple. It has its own process to classify and assign the patient to the right place at the right time. It has two consecutive stages. The primary stage is collecting data which enable nurses to assign patients to different health departments; and the second stage is initiation of nursing care at the emergency department to prevent premature death and decrease further complications [3].

The most important device to achieve the desired health outcome is the right triage, which is made by nurses. Because, the doctors can get his or her client at the right time that enables them to make fruitful treatment. On the other hand, nurses may give high attention to the a patient actually needs less attention or they may give less attention to the patient who needs high attention [3].

The triage nurse in the emergency department is the first person that a patient encounter when presenting for emergency care of the department. The triage nurses’ skill has been as influential factors in triage decision-making [4, 5].

Triage skill among nurses is the key element of supervision of emergency department. If it is not carried out at standard level, the outcomes of clinical care of patients and efficiency of the emergency departments get compromised [6].

Even though, valid and reliable triage acuity is very crucial, nurses’ level of triage skill is still at moderate level [7].

A study conducted in Indonesia showed that most of the participants (65.40%) perceived their overall triage skill is at a moderate level, and they felt the same towards each of the sub-dimensions of triage skill [7].

Despite understanding the level of nurses’ triage skill and associated factors is very critical issue to design appropriate interventions, no study has been done in Ethiopia on this topic yet.

Therefore, the aim of this study was to assess the level of nurses’ triage skill and associated factors among emergency nurses in Addis Ababa.

## Main text

### Methods

#### Study setting, design, period and participants

An institutional based cross-sectional study was conducted at public hospitals in Addis Ababa from 1st April to 1st May 2017. Addis Ababa, the capital city of Ethiopia has 39 hospitals (12 public and 27 private), 29 health centers, 122 health stations, 37 health posts and 382 modern private clinics. Out of 12 public hospitals, 2 specialized hospitals provide specific psychiatric and Obstetric services. The rest ten hospitals provide general emergency service with 333 emergency nurses. All nurses working in emergency department of public hospitals were included in this study.

#### Sample size determination and sampling procedure

The sample size was calculated using single population proportion formula by considering the following assumptions: 50% proportion (p) to get maximum sample size, 95% level of confidence (CL), 5% margin of error (d) and 10% non-response rate and the sample size were 384 nurses, but since the total study population was less than 10,000, correction formula was made to get the actual sample size which was 197 nurses.

$${\text{n}} = \frac{{\left( {{\text{Z}}\upalpha/ 2} \right)^{ 2} {\text{P}}\left( { 1- {\text{P}}} \right)}}{{{\text{d}}^{2} }}$$

where, n is the sample size, p is the proportion of triaging skills amongst emergency nurses (50%), Z is the standard normal distribution curve value for the 95% confidence interval (1.96), d is the the margin of error or accepted error

$${\text{n}} = \frac{{\left( { 1. 9 6} \right)^{\text{2}} *0. 5\left( { 1- 0. 5} \right)}}{{\left( {0.0 5} \right)^{ 2} }}$$

n = 384 nurses working in emergency department.

Since the source population was 333 and less than 10,000, correction formula was used to get the actual sample size as follows

$${\text{nf}} = {\text{ n}}/\left( { 1+ {\text{n}}/{\text{N}}} \right)$$

where, nf is the Final sample size, n is the first calculated sample size, N is the Source population, n final is the 384/(1 +384/333) = 179.

Finally, adding 10% of a non-response rate, the total sample size was = 197 emergency nurses.

Each hospital has a different number of nurses. So to reach to each individual, first the sample size was proportionally allocated to each hospital. Then, simple random sampling technique was used to select participants using the lottery method from each hospital.

#### Measurements

A pre-tested structured self-administered questionnaire was used to collect data from study participants. The questionnaire was constructed by review previous studies done on similar topics. It has two parts: demographic data sheet (DDS) and triage skill questionnaire (TSQ). Triage skill was measured by using a triage skill questionnaire. A triage skill questionnaire (TSQ) was a 37-item questionnaire with three dimensions, including rapid assessment, patient categorization, and patient allocation. Participants were asked to respond to each item using 1–5 rating scale: 1 = need improvement, 2 = poor, 3 = fair, 4 = good, and 5 = very good. The possible range of the total score of triage skill was 37–185. The total score was converted to a percentage. Using the criterion referenced, the score was interpreted as 60% = low level of triage skill, 60–80% = moderate level of triage skill, and > 80% = high level of triage skill. The content validity of the questionnaire was evaluated by three experts in Indonesia and the Cronbach’s alpha coefficient was .93 [7] (see Additional file 1).

#### Statistical analysis

After data collection, each questionnaire was checked for completeness. Data was coded, cleaned, and analyzed by using (SPSS) version 22.0. The data were cleaned for inconsistencies and missing values. Simple frequencies were used to see the overall distribution of the study subject with the variables under study. A Pearson correlation was used to determine the association between factors and the outcome variable. Those variables which had significant association with skill of triaging among nurses had been entered into multivariate linear regression analysis.

### Results

#### Socio-demographic characteristics of the respondents

One hundred eighty-nine nurses were participated in this study, which made response rate of 95.4%. Around half of the participants (51.9%) were females. Nearly two-third (63.8%) of the respondents were single. Most of the respondents (74.1%) had educated of first degree in nursing.

About 49.2% of the respondents had working experience of less than 1 year in the emergency department with a mean 21.30 months (SD = 15.91). Only 19.6% participants worked in the triage room with a minimum of 1 month and maximum of 48 months with a mean of 12.18 months (SD = 10.68).

Around 49.2% of the respondents had attended at least one training in the past 3 years with mean training index of 5.13 (SD = 8.48) (Table 1).

**Table 1:** Socio demographic characteristics of nurses working in the emergency departments in Addis Ababa public hospitals, Ethiopia, 2017 (n = 189)

Characteristic	Category	Frequency	Percent
Sex	Male	91	48.1
	Female	98	51.9
Age	< 20	3	1.6
	20–30	147	77.7
	31–40	31	16.4
	> 40	8	4.3
Marital status	Married	69	36.5
	Unmarried	120	63.5
Religion	Orthodox	85	45.0
	Protestant	50	26.4
	Muslim	40	21.2
	Other	14	7.4
Ethnicity	Oromo	56	29.6
	Amhara	73	38.6
	Tigre	41	21.7
	Gurage	5	2.6
	Other	14	7.5
Educational level	Diploma	30	15.8
	BSc degree	140	74.1
	MSc degree	19	10.1
Work experience in emergency department	Less than 1 year	93	49.2
	3–5 years	72	38.1
	Greater than 5 years	24	2.1
Work experience in triage unit	Yes	37	19.6
	No	151	80.4
Trainings taken	Yes	93	49.2
	No	96	50.8
Type of trainings taken	Infection prevention training	31	33.0
	25.4% had attended the Triage Officer Course	27	25.4
	Basic Trauma Life Support (BTLS) training course	20	21.2
	Trauma in nursing care	14	14.3

*ED* emergency department

#### Level of triage skill of nurses

Above half of the respondents (52.9%) had a moderate level of triage skill (Additional file 2).

#### Factor analysis of triage skill using Pearson correlation

Strong, positive relationships were found to exist between triage skill and triage knowledge (r = .68, p .01) and a moderate positive relationship exist between triage skill and the three other factors: training experience (r = .56, p .01), work experience in emergency department (r = .52, p .01), and work experience in the triage room (r = 0. 49, p .01) (Table 2).

**Table 2:** Associated factors of triage skill among nurses working in the emergency department of Addis Ababa public hospitals, Ethiopia, 2017

Variables	N	Pearson correlation	Sig. (2-tailed)
Triage knowledge	189	.68**	< .001
Training experience	93	.56**	< .001
Work experience in ED	189	.52**	< .001
Work experience in triage room	37	.48**	< .001
Level of education	189	.47**	< .001

*ED* emergency department, *N* number

** Correlation is significant at the .01 level (2-tailed)

#### Factor analysis of triage skill using multivariable linear regressions

Univariate linear regression was used to assess variables, whether they had influence over triage skill of nurses working in the emergency department. In the univariate linear regression analysis, factors such as: knowledge about triage, training, work experience in the emergency department, work experience in triage room and educational level had significant relationship with triage skill. Variables which were significant at univariate linear regression were entered in multivariable linear regression to control confounding effect.

Finally, training experience, knowledge about triage, and educational level had significant relationship with triaging skill.

Knowledge about triage [B = 1.09, CI (1.41, 1.77), p = .002] was positively associated with triage skill. This association tells us in every unit increase in triage knowledge, there is around 1.09 unit increases in triaging skill.

There is also a significant association between level of education and triage skill. Diploma, emergency nurses had around 19.96 times lower triage skill when compared to nurses who had a degree [B = − 19.96, CI (− 30.208, − 9.715), p = .001].

Training experience had significant association to triage skill [B = .55, CI .16, .94), p = .006] (Table 3).

**Table 3:** Multivariable linear regression model showing associated factors of triage skill among nurses working in the emergency department of Addis Ababa public hospitals, Ethiopia, 2017

Variables entered into model	Unstandardized coefficients		Standardized coefficients	*p* value	95% confidence interval for B	
	B	Std. error	Beta		Lower bound	Upper bound
Work experience in ED	.13	.14	.11	.350	− .14	.40
Work experience in triage room	.08	.19	.04	.703	− .32	.46
Training about triage	.55	.19	.26	*.006*	.16	.94
Triage knowledge Education level BSc*(reference)	1.09	.35	.27	*.002*	1.41	1.77
MSc	3.98	4.68	.07	.396	− 5.28	13.25
Diploma	− 19.96	5.18	− .26	*.001*	− 30.21	− 9.72

Dependent variable = triage skill

*ED* emergency department

* Variables which are candidate for multiple linear regression (< .05) were included in the model. R^2^ = .54

The italic value shows the significant values

### Discussion

This study revealed that greater than half of the nurses had a moderate level of triage skill. This result is comparable to the study conducted in Dar Es Salaam, Tanzania 52% [5], and study conducted in Indonesia that the mean score of triage skill and its sub-dimensions were at a moderate level [7]. But the result is lower than the studies done in Indonesia 65.4% [7], Sweden 60.3% [8], and Switzerland 59.6% [9].

The difference might be due to the variation in sample size and study settings. For instance, the Indonesian study included 266 nurses from two secondary and two tertiary hospitals. While our study selected 197 nurses from one tertiary and nine secondary hospitals. Working in tertiary hospital has more chance to be exposed to more complicated and variety of cases. This challenge can enforce institutions and nurses to develop a skill to handle those cases.

In case of studies done in Switzerland and Sweden, the possible discrepancy might be due to sample size and tool difference. In those two studies the sample size and the tool were 69,423 nurses and Emergency Severity index and Canadian Triage and Acuity scale were used respectively. But, in the current study, a triage skill questionnaire was used.

In addition to this, the difference might from curriculum vary across different countries, hospital set ups and training experience among professionals. In Ethiopia, emergency nursing speciality was started in recent years. As a result, almost all nurses working in the emergency department were comprehensive nurses.

Training about triage also can be another reason for the difference, among the participants of this study only a quarter of them had attended training about triage. On the other hand, nurses who have attended training about triage can fill the gap they have and this in turn increases the skill of triage.

The other possible reason for this difference finding might be an economic factors, since Ethiopia is the developing country, a scarce resource for nursing students during their study, over crowdedness of the available hospitals, and lack of basic infrastructures for the hospitals might hinder the practitioners to prioritize the clients in the emergency room.

In this study triage skill of nurses was influenced by triage training, knowledge about triage, and educational level. The result is in line with different studies [7, 10–12].

This may be due to the fact that an increase in educational level, training, and knowledge increases skill acquisition. When a nurse understands the urgency, severity, and outcome of the problem early, it is easy for them to triage clients immediately. Continues trainings and higher educational levels are directly a means to increase knowledge and skill of nurses in a diverse aspect of situations which prepare them psychologically as well as mentally to respond emergency situations.

### Conclusion

The findings provide a better understanding of triage skill among emergency nurses in Addis Ababa, Ethiopia. The triage skill was found to be at a moderate level. In addition, There were significantly positive relationship between triage skill and triaging knowledge, training, and educational level. Therefore, the ministry of health and hospitals should provide trainings and education to improve triage skill.

## Limitation

This study had a limitation in terms of convenience sampling used and conducted in an urban area which limits generalizability of findings and it assessed only the quantitative aspects of triage skill and this may overestimate the actual level of triage in the study area due to recall bias. The other limitation of this study was the use of a triage skill questionnaire that was not valid in Ethiopia.

## Additional files

**Additional file 1.** Triage questionnaires. A Triage Skill Questionnaire (TSQ) was a 37-item questionnaire with three dimensions, including rapid assessment, patient categorization, and patient allocation. Participants were asked to respond to each item using 1-5 rating scale: 1 = need improvement, 2 = poor, 3 = fair, 4 =good, and 5 = very good. The possible range of the total score of triage skill was 37-185. The total score was converted to a percentage. Using the criterion referenced, the score was interpreted as 60% = low level of triage skill, 60-80% = moderate level of triage skill, and > 80% = high level of triage skill.

**Additional file 2.** Distributions of levels of triage skill among nurses working in the emergency department in Addis Ababa public hospitals, Ethiopia, 2017. The figure shows the distribution of levels of triage skill among nurses by dividing in to three levels. About 10.1%, 52.9, and 37% of nurses had low, moderate and high level of triage skill respectively.

## Abbreviations

ED: emergency department
DDS: demographic data sheet
TSQ: triage skill questionnaire
