# Supplementary material for: Triage skill and associated factors among emergency nurses in Addis Ababa, Ethiopia 2017: a cross-sectional study
Source: BMC Res Notes. 2018 Sep 10;11:658. doi: 10.1186/s13104-018-3769-8 (PMC6131741; doi:10.1186/s13104-018-3769-8)
Supplement: Supplementary file 1 — Additional file 1. Triage questionnaires. A Triage Skill Questionnaire (TSQ) was a 37-item questionnaire with three dimensions, including rapid assessment, patient categorization, and patient allocation. Participants were asked to respond to each item using 1-5 rating scale: 1 = need improvement, 2 = poor, 3 = fair, 4 =good, and 5 = very good. The possible range of the total score of triage skill was 37-185. The total score was converted to a percentage. Using the criterion referenced, the score was interpreted as 60% = low level of triage skill, 60-80% = moderate level of triage skill, and > 80% = high level of triage skill. [file 13104_2018_3769_MOESM1_ESM.docx]

**QUESTIONNAIRE TO ASSESS KNOWLEDGE OF TRIAGE AMONG NURSES WORKING IN THE EMERGENCY DEPARTMENTS IN DAR ES SALAAM:**

**Demographic data**: Please complete the following questions

(Tick the appropriate)

Questionnaire #.........

1. Where are you currently working?

1. Muhimbili national hospital……………
2. Mwananyamala hospital……………….
3. Amana hospital………………………...
4. Temeke hospital……………………….

2. What is your level of nursing education?

(a) Certificate ……………..

(b) Diploma………………...

(c) Advanced diploma……….

(d) BScN……………..

3. Postgraduate/post basic nursing training:

(a) Certificate in emergency/trauma care……………..

(b) Certificate in critical/intensive care………………

(c) Other certificate (specify)…………………………

4. How long have you been working in the emergency department?

(a) Below 1 year……………………

(b) Between 1 to 3 years…………...

(c) Between 3 to 5 years…………...

(d) Between 5 to 10 years………….

(e) Others (specify)………………...

5. Have you ever been trained on emergency nursing care?

(a) Yes………..

(b) No………..

6. If **YES** on question 6, did the training include how to triage patients? :

(a) Yes………

(b) No……….

**Knowledge of triage: this section assessed the participants’ knowledge of triage.**

1. Define triage ...................................................................................................................................................................................................................................................................................
2. How long should a patient with the following color code wait for treatment in the casualty or emergency unit.(select your answer from here: less than 10min, immediate, less than 60 min, less than 240min)
   1. Red………………………………..
   2. Orange…………………………….
   3. Yellow……………………………..
   4. Green……………………………..

**Assume you are at the casualty or emergency unit as a triage nurse. Assign priorities to the following patients who come for emergency health care services with the following conditions. (*Label as P1, P2, P3: where P1 stands for first priority, P2 second priority, and P3 third priority*)*.***

1. An Adult patient. Breathing with difficult. Respirations of 8/min, Systolic blood pressure of 80mmHg……………….
2. An Adult patient. Fast breathing. Coughing. Respirations of 40/min, Temperature of 39 degrees centigrade …………………
3. An Adult patient. Face burn. On severe pain. Respirations of 29, Pulse rate of 129/min…………
4. Female. Pregnancy. walking. Trauma. Respirations of 20/min, Pulse rate of 100/min, and Systolic blood pressure of 130mmHg………………..
5. Adult patient. Walking. Feeling discomfort. Headache. Systolic blood pressure 200mmHg………………….
6. Adult patient. Skin moderate pale, cool, and dry. Reacts to voice. Respirations of 28/min, pulse rate of 110/min, Systolic blood pressure of 90mmHg………………..
7. Adult patient on stretcher. Controlled bleeding. Weak peripheral pulse. Respirations of 20/min, pulse rate of 100/min, Systolic blood pressure of 130mmHg………………….
8. Adult patient with chest pain radiating to the left arm. Walking. Respirations of 30/min, pulse rate of 110/min, Systolic blood pressure of 90mmHg……………

**NURSING TRIAGE OBSERVATION checklist.**

**The checklist was used to assess triage practices for nurses working in the emergency departments by observing the participants while they were performing their triage activities:**

- Hospital…………………………….

|  | Items to be checked | response | | |
| --- | --- | --- | --- | --- |
|  |  | Yes | No | Comments |
| 1 | Is there a nurse assigned for patients’ triage |  |  |  |
| 2 | If Yes in 1 above, does the nurse actually triage the patients |  |  |  |
| 3 | Does the triage performance involve: |  |  |  |
|  | - air way assessment |  |  |  |
|  | - respiratory status assessment |  |  |  |
|  | - circulatory status assessment |  |  |  |
| 4 | Does respiratory status assessment includes: |  |  |  |
|  | - look for chest movement |  |  |  |
|  | - Listen for breath sound |  |  |  |
|  | - Breathing pattern |  |  |  |
| 5 | Does circulatory status assessment includes: |  |  |  |
|  | - Blood pressure |  |  |  |
|  | - Pulse rate |  |  |  |
|  | - skin/mucous color (pink, pale) |  |  |  |
|  | - Skin temperature (warm, hot, cool/cold) |  |  |  |
|  | - Capillary refills |  |  |  |
| 6 | Temperature measurement |  |  |  |
| 7 | Pain assessment |  |  |  |
| 8 | Neurological assessment: Alert, responds to voice, responds to pain, Unresponsive. |  |  |  |
| 9 | Does the nurse document the triage anywhere? |  |  |  |
| 10 | Does re-assessment on regular basis of those patients who are waiting done to see if their conditions have deteriorated and need to be seen more urgently? |  |  |  |
| 11 | If No in question 1 above, how patients are handled in the emergency departments |  |  |  |
|  |  |  |  |  |
|  |  |  |  |  |
|  |  |  |  |  |
|  |  |  |  |  |
|  |  |  |  |  |

**EQUIPMENTS REVIEW GUIDE:**

**This tool was used to assess the presence of equipment’s that assist nurse in performing triage.**

- Hospital:………………………………………………

1. Presence of triage guidelines (e.g. triage scale and acuity rating, color code) for rating the acuity

of patients in the hospital emergency department: (*tick the appropriate answer*).

(a) Yes………….

(b) No…………..

2. If the answer is **NO** for question 1 above, how does the patient’s category and acuity is rated: ………………………………………………………………………………………………………………………………………………………………………………………………………………………………………………………………………………………………………………………………………………………

4. Does the triage guidelines states what actions to be taken and within which duration of time?

(a) Yes…………………….

(b) No………………………

5. Presence of facilities in the hospital emergency department that assists nurse in performing

triage assessment :(*tick all available )*.

(a) thermometer…………………………………………….

(b) pulse oxymeter………………………………………….

(c) sphygmomanometer…………………………………….

(d) stethoscope……………………………………………..

(e) glucometer and its strips………………………………..

(f) urinalysis strips………………………………………….

(h) 12 lead electrocardiography……………………………..

(i) ABG machine and its reagents………………………….

(j) observational chart (including level of illness severity)…………..

(k) pain assessment scale……………………………………………..

**APPENDIX C**

**Triage Skill Questionnaire (TSQ)**

Instruction: Please assess your own ability in triage skills by check list the number on scale 1 to 5 following each statement below. There are five options available: **5 = very good, 4 = good, 3 = fair, 2 = poor, 1 = need, improvement**

| **No** | **Triage skills** | **Perceived Triage Skill** | | | | |
| --- | --- | --- | --- | --- | --- | --- |
|  |  | Very good  (5) | Good  (4) | Fair  (3) | Poor  (2) | Need improvement  (1) |
|  | **Rapid Patient Assessment** |  |  |  |  |  |
| **1** | Aassess patient include vital signs with rapid assessment in 2-5 minutes |  |  |  |  |  |
| **2** | Aassess or ask chief complaint of the patient rapidly |  |  |  |  |  |
| **3** | In unconscious patients, look in the upper airway such as blood, vomit, foreign bodies, oedema, and tongue obstruction as assess airway patency |  |  |  |  |  |
| **4** | Decide to open airway and remove foreign body when airway is obstructed according to airway management (A) |  |  |  |  |  |
| **5** | Give positioning airway to maintain patency by chin lift |  |  |  |  |  |
| **6** | Perform clear airway by correct position with jaw trust and head tilt |  |  |  |  |  |
| **7** | Perform clear airway by correct position by jaw trust without head tilt if the patient suspect cervical spinal |  |  |  |  |  |
| **8** | Perform to insert oropharyngeal or nasopharyngeal airway |  |  |  |  |  |
| **9** | Look at the chest about patient chest abnormal movement |  |  |  |  |  |
| **10** | Assess rate and depth of respirations to observe (B) breathing rate and pattern rhythm with look and listen |  |  |  |  |  |
| 11 | Look at the patient skin to investigate for integrity, wounds, bruising, texture, and color |  |  |  |  |  |
| 12 | Listen the noise in the airway such as gurgling, snoring, and wheezing |  |  |  |  |  |
| 13 | Listen the silent or noisy breathing |  |  |  |  |  |
| 14 | Feel air blow from the patient with my cheek |  |  |  |  |  |
| 15 | Administer oxygen therapy |  |  |  |  |  |
| 16 | Perform manual ventilation |  |  |  |  |  |
| 17 | Perform bag-valve-mask ventilations |  |  |  |  |  |
| 18 | Protect cervical spine when patient suspect fracture cervical with cervical collar |  |  |  |  |  |
| 19 | Check pulse rate and rhythm according circulation assessment (C) |  |  |  |  |  |
| 20 | Assess of the capillary refill |  |  |  |  |  |
| 21 | Assess the temperature the patients |  |  |  |  |  |
| 22 | Assess the patient with diaphoresis |  |  |  |  |  |
| 23 | Perform chest compressions in critical condition of the patient |  |  |  |  |  |
| 24 | Collaborative with physician to administer emergency drugs |  |  |  |  |  |
| 25 | Assess internal and external bleeding |  |  |  |  |  |
| 26 | Perform control blood loss appropriately to stop bleeding the patient |  |  |  |  |  |
| 27 | Collaborate resuscitation to provide appropriate intravenous fluid |  |  |  |  |  |
|  | **Patient categorization** |  |  |  |  |  |
| 28 | Catagorization the patient according to triage priority |  |  |  |  |  |
| 29 | Identify the patient who require immediate care, urgent, and non urgent according to triage categorization |  |  |  |  |  |
| 30 | Avoid the condition of the patient with over-triage and under-triage |  |  |  |  |  |
| 31 | Initiation nursing intervention during triage categorization |  |  |  |  |  |
|  | **Patient allocation** |  |  |  |  |  |
| 32 | Make a decision to allocate the patient with priority 1 (Resuscitation in ED) in the right place |  |  |  |  |  |
| 33 | Make a decision to allocate the patient with priority 2  (Critical care in ED) in the right place |  |  |  |  |  |
| 34 | Allocate make a decision to allocate to the patient with priority 3 in the right place  (Ambulatory in ED) correctly |  |  |  |  |  |
| 35 | Allocate the patient with nursing intervention safety in ED |  |  |  |  |  |
| 36 | Allocate the patient by collaboration with other emergency nurse & medical doctor with hand over effectively |  |  |  |  |  |
| 37 | Allocate the patient to get advance treatment in ED in accurately and timely |  |  |  |  |  |
