# Supplementary material for: Triage skill and associated factors among emergency nurses in Addis Ababa, Ethiopia 2017: a cross-sectional study
Source: BMC Res Notes. 2018 Sep 10;11:658. doi: 10.1186/s13104-018-3769-8 (PMC6131741; doi:10.1186/s13104-018-3769-8)
Supplement: Supplementary file 2 [file 13104_2018_3769_MOESM2_ESM.docx]

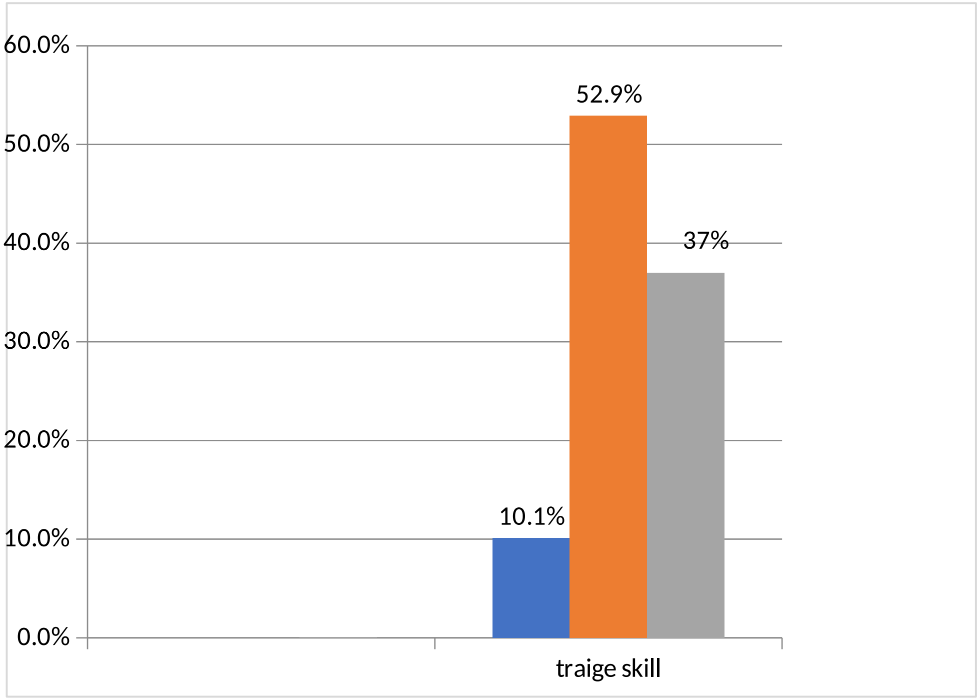
60.0%
50.0%
40.0%
30.0%
20.0%
10.0%
0.0%
52.9%
37%
10.1%
traige skill
